# Influence of Carbon Nanotubes on the Mechanical Behavior and Porosity of Cement Pastes Prepared by A Dispersion on Cement Particles in Isopropanol Suspension

**DOI:** 10.3390/ma13143164

**Published:** 2020-07-15

**Authors:** Vanessa Vilela Rocha, Péter Ludvig

**Affiliations:** Department of Civil Engineering, Federal Center for Technological Education of Minas Gerais, Belo Horizonte, Minas Gerais 30.421-169, Brazil; peter@cefetmg.br

**Keywords:** carbon nanotubes, dispersion, cement

## Abstract

Cement composites prepared with nanoparticles have been widely studied in order to achieve superior performance structures. The incorporation of carbon nanotubes (CNTs) is an excellent alternative due to their mechanical, electrical, and thermal properties. However, effective dispersion is essential to ensure strength gains. In the present work, cement pastes were prepared incorporating CNTs in proportions up to 0.10% by weight of cement, dispersed on the surface of anhydrous cement particles in isopropanol suspension and using ultrasonic agitation. Digital image correlation was employed to obtain basic mechanical parameters of three-point bending tests. The results indicated a 34% gain in compressive strength and 12% in flexural tensile strength gains, respectively, as well as a 70% gain in fracture energy and 14% in fracture toughness in the presence of 0.05% CNTs were recorded. These results suggest that CNTs act as crack propagation controllers. Moreover, CNT presence contributes to pore volume reduction, increases the density of cement pastes, and suggests that CNTs additionally act as nucleation sites of the cement hydration products. Scanning electron microscopy images indicate effective dispersion as a result of the methodology adopted, plus strong bonding between CNTs and the cement hydration product. Therefore, CNTs can be used to obtain more resistant and durable cement-based composites.

## 1. Introduction

Carbon nanotubes (CNTs) have been attracting the attention of the scientific community over the last few decades due to their extraordinary mechanical, thermal, and electrical properties. The CNTs’ mechanical properties, such as high tensile strength and Young’s modulus, are the principal reasons for their use as composite materials [1]. In order to achieve effective composite preparation, good dispersion and homogeneous distribution of CNTs are essential. The hydrophobic behavior causes CNT agglomerations and the clusters formed by their presence in high amounts can compromise the mechanical properties [2,3,4]. In addition, due to this characteristic, the nanostructured cement composites’ strength presents high variability [2], and consequently, reproducibility and statistical analysis are important to the validation of results.

Studies on CNTs in cement-based materials have suggested improvements in mechanical properties of cement pastes [5,6,7,8,9,10,11], including reduction of porosity [4,10], reduction of early shrinkage [11], an increase in the fracture energy [11,12], and an increase in fracture toughness and proposal that the presence of well-dispersed CNTs in the cement matrix makes it necessary to apply higher stress to cause cracking and specimen rupture [6]. Moreover, CNTs contributed to a refinement of the mesopores and resulted in a denser matrix [13,14]. Cement paste, with up to 0.20% of CNTs dispersed in aqueous surfactant solution by sonication, indicated that the quantity of macropores (diameter greater than 50 nm) was reduced, while the quantity of pores smaller than 50 nm was increased [15]. Macropores in hardened cement composites are considered to be detrimental as they cause worse mechanical properties and lower density of these materials [15]. Mortars prepared with CNTs dispersed in an aqueous solution with superplasticizers registered a reduction in the quantity of micropores (less than 2 nm) and macropores (greater than 50 nm). It is alleged that this pore refinement on a micro and macro scale is more relevant to obtain gains in mechanical strength than reducing the total porosity [16].

With the aim to increase the dispersion efficiency of CNTs, nanomaterial can be submitted to chemical treatments, which is called functionalization. Functionalization occurs through noncovalent (weak bonds with CNTs) and covalent (strong interactions, usually with significant modifications on CNT properties) interactions [17]. Functionalization treatments result in good dispersion of CNTs in aqueous solutions [18], however, researchers suggest that some types of functionalization can damage the carbon nanotubes structure [19,20].

According to Alsharefa et al. (2017) [21], dispersion through chemical mixtures damaged the structure of CNTs and studies involving physical dispersion should be intensified. Thereby, an option of a non-functionalized methodology through non-aqueous suspensions has been adopted for effective dispersion of CNTs in cement particles [1,22]. Isopropanol, which is a less polar solvent as compared with water, is capable of promoting superior CNT dispersion on the surface of cement particles. However, it does not cause any hydration reaction in Portland cement. The dispersion is enhanced by the employment of ultrasonic frequency agitation and mechanical shaking. Isopropanol is removed thereafter, followed by the preparation of the cement paste. This process can be considered much simpler in execution than the use of covalent functionalization. At the same time, the advantages of this methodology are evidenced by the densification of hydrated calcium silicates (C–S–H) [22], which have the major responsibility for the final strength of cement-based materials. Furthermore, this dispersion process provides an indication of a strong connection between CNTs and hydrated cement matrix, controlling the crack propagation [23]. The dispersion of similar nanomaterial, i.e., multilayer graphene, in isopropanol results in adequate dispersion, avoiding multilayer graphene agglomeration and recording improvements in mechanical properties of the composites [24]. The nanocomposite produced with multilayer graphene shows approximately 100% higher splitting tensile and compressive strengths.

This present article aims to evaluate the effect of adding CNTs on the mechanical properties of cement. In order to achieve the objectives, predispersions in isopropanol media of CNTs on cement particles were prepared and used to evaluate the physical and mechanical behavior of cement pastes incorporating 0%, 0.05%, and 0.10% of CNTs according to cement weight. Using these proportions and methodology, Rocha and Ludvig (2018) [9] achieved approximately 50% gain in compressive and splitting tensile strengths at 0.05% of CNTs, suggesting that the optimum range for incorporation of CNTs based on that methodology is close to this ratio. Hawreen et al. (2018) [3] also confirmed that amounts of CNTs, up to 0.10%, were more effective for increasing the flexural and compressive strengths.

## 2. Materials and Methods

The cement pastes were prepared without additives or admixtures, and the CNTs did not undergo any type of functionalization. At 28 days, the cement pastes were mechanically characterized regarding their compressive strength, flexural tensile strength, fracture energy, and fracture toughness. Moreover, their void index and pore distribution were determined, as well as their thermogravimetric analysis was performed.

### 2.1. Materials

The materials used in this research were:

- Multiwalled carbon nanotube (MWCNT) with estimated tube lengths between 5 and 30 μm, 99% of external diameter between 10 and 50 nm, and purity higher than 93%, produced by CTNano, Belo Horizonte, Minas Gerais, Brazil;
- Brazilian type CP-V Portland cement because of its low percentage of mineral additions;
- Isopropanol absolute grade;
- Potable water for cement hydration.

### 2.2. Dispersion Process

Two different formulations with CNTs were carried out to prepare the cement paste, i.e., adding 0.05% and 0.10% by total weight of cement (bwoc).

**Figure 1:**
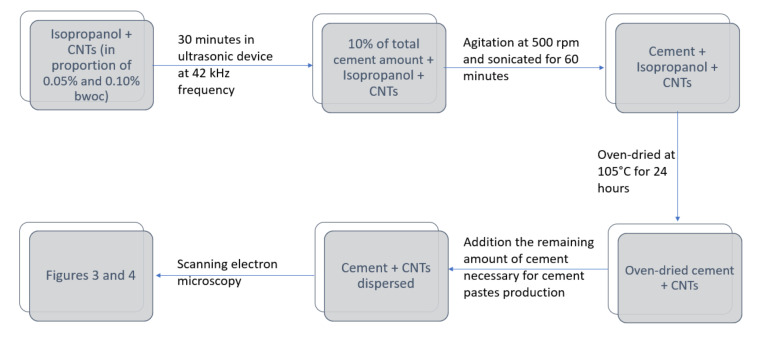
The adopted methodology to disperse carbon nanotubes (CNTs) in a suspension of isopropanol.

The CNTs were dispersed in non-aqueous isopropanol solution. The steps for dispersing CNTs in anhydrous cement particles are described in Figure 1.

The scanning electron microscopy images were acquired using a FEG Quanta 200 made by FEI Company (located at the Center of Microscopy, UFMG, Belo Horizonte, Minas Gerais, Brazil) to evaluate the CNT dispersion. To ensure the electrical conduction on the samples, a 5 nanometers thick carbon coating was applied.

The surface area of the anhydrous cements with dispersed CNTs with concentrations of 0%, 0.05%, and 0.10%, were also determined by nitrogen adsorption using multipoint technique.

### 2.3. Evaluation of the Mechanical Behavior

The cement pastes were prepared in a mortar blender with 0.33 water/cement ratio. For each formulation, 4 prismatic specimens with 4 cm height, 4 cm width, and 16 cm length for flexural tensile test were prepared. The specimens were maintained in lime-saturated water until they reached 28 days, and then tested. The formulations analyzed are described in Table 1.

Prior to the three-point flexural tests, a 1 cm deep notch was made in the middle of the bottom of the beams. The cut was performed in a special apparatus to ensure alignment. In sequence, the lateral sides of the test samples were painted with white and black spray to create a stochastic pattern in each sample to guarantee the digital image correlation (DIC) analysis efficiency. The tests were conducted on a universal mechanical testing machine equipped with 5 kN load cell at a loading speed of 0.25 mm/min.

The flexural test was photographed throughout the loading at 250 milliseconds intervals. Using the sequence of high-resolution and accurate photos, a correlate software recorded the horizontal and vertical displacements suffered during the test, as well as the applied force. The results obtained were used to calculate the fracture energy (*G*_f_) and fracture toughness (*K*_IC_) of the samples. These two parameters were calculated according to the formulation indicated by Dally and Riley (1991) [25] and by Hu et al. (2014) [6] as indicated in the equations below:

(1) $$Gf=\frac{m\;g\;\delta +W}{t\;\left(h-a\right)}\;$$

(2) $$K_{IC}=\frac{P\;S}{t\;h^{\frac{3}{2}}}\;f\left(\frac{a}{h}\right)\;$$

(3) $$f\left(\frac{a}{h}\right)=2.9{\left(\frac{a}{h}\right)}^{\frac{1}{2}}-4.6{\left(\frac{a}{h}\right)}^{\frac{3}{2}}+21.8{\left(\frac{a}{h}\right)}^{\frac{5}{2}}-37.6{\left(\frac{a}{h}\right)}^{\frac{7}{2}}+38.7{\left(\frac{a}{h}\right)}^{\frac{9}{2}}$$

where *G_f_* is the fracture energy of the composite; *K*_IC_ is the fracture toughness of the composite, *W* is the area under the load-displacement curve, *m* is the mass of the beam between supports, *g* is the acceleration due to gravity, *δ* is the vertical displacement at final failure of the beam, *t* is the width of the specimen, *h* is the height of the specimen, *a* is the depth of the slot, *S* is the span of the beam, *P* is the peak load, and *f(a/h)* is the geometry factor [25].

The results of the flexural tensile strength tests were also compared with the results obtained by Rocha and Ludvig (2018) [9] realized using a splitting tensile strength test.

**Figure 2:**
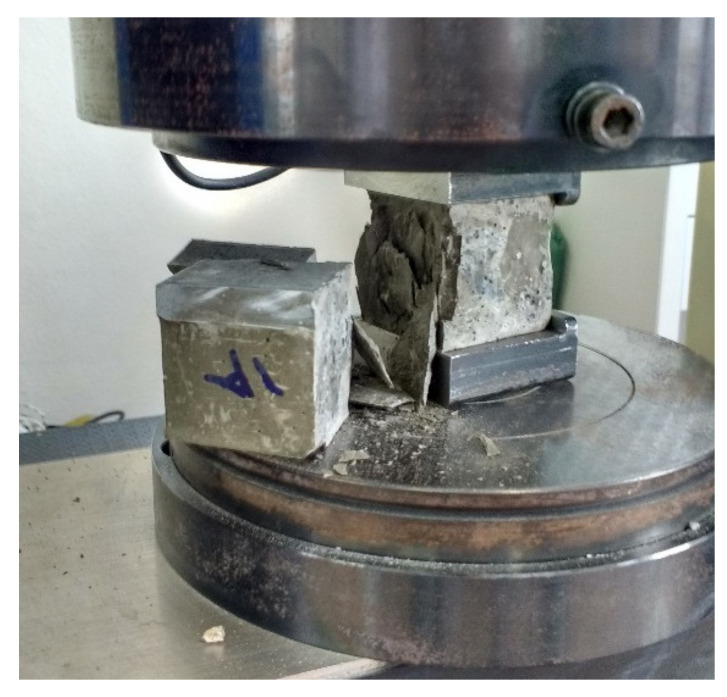
Setup and specimen failure of the compressive strength test.

After the three-point flexural test, one of the remaining halves of the prismatic samples was used to perform compression tests as an adaptation of ASTM C349-02 [26]. The fragments were placed between two metal plates with an area of 4 cm × 4 cm, as indicated in Figure 2. The sample was submitted to continuous loading in the EMIC brand universal equipment located at the CTNano test laboratory, using a 200 kN load cell and load speed of 0.50 mm/sec. The compressive strength results were compared with the results obtained by Rocha and Ludvig (2018) [9].

It is noteworthy that Rocha and Ludvig (2018) [9] analyzed the mechanical properties based on compressive and splitting tensile tests on 5 cm diameter and 10 cm height cylindrical samples. In the present work, the tensile strength was assessed by flexural test in prismatic samples and the compressive strength was evaluated on the remaining halves of the prismatic samples, as exhibited in Figure 2. Complementing previous work [9], we performed a microstructural analysis of the cement paste to justify the results of mechanical strength, likewise, the mechanical properties analysis was developed according to a different methodology.

### 2.4. Evaluation of the Physical Parameters

The other half of the prismatic sample was used to determine density in the saturated and dry conditions, void index, and water absorption as follows:

- The samples were dried at 105 °C for 72 h, cooled to room temperature for 30 min, and weighed, obtaining the dry mass (*M*_S_);
- Then, the samples were completely immersed in water and maintained for 72 h;
- After this period, the masses of the submerged saturated sample (*M*_i_) and saturated sample with dry surface (*M*_SAT_) were measured.

These indexes were used for the calculations using the follow [27]:

(4) $$Water\;absorption=\frac{M_{SAT}-M_{S}}{Ms}\times 100$$

(5) $$Void\;index=\frac{M_{SAT}-M_{S}}{M_{SAT}-M_{i}}\times 100$$

(6) $$Density\;of\;dried\;samples=\frac{M_{S}}{M_{SAT}-M_{i}}$$

(7) $$Density\;of\;saturated\;samples=\frac{M_{SAT}}{M_{SAT}-M_{i}}$$

Fragments of each paste formulation were subjected to a pore size distribution analysis using nitrogen condensation technique based on DFT (density functional theory), a quantitative assessment of the composition by thermogravimetric analysis, and scanning electron microscopy in order to evaluate the microstructure morphology.

To determine the pore distribution by nitrogen condensation, the cement pastes samples were degassed for 24 h in a vacuum at 30 °C [28]. The analysis was performed using 20 adsorption and 20 desorption points. The pore size distribution was analyzed using the DFT method based on desorption data.

Whereas, to perform the thermogravimetric analysis, the paste fragments were ground and sieved and only the particles smaller than 75 micrometers were subjected to analysis. The analysis was carried out until reaching 1000 °C with a heating rate of 10 °C/min in a synthetic air environment on a Shimadzu Corporation DTA-60H thermal analyzer (located at the CTNano, Belo Horizonte, Minas Gerais, Brazil).

The scanning electron microscopy images were captured using a FEG Quanta 200 FEI equipment (located at the Center of Microscopy, UFMG, Belo Horizonte, Minas Gerais, Brazil) for the hydrated cement pastes with concentrations of 0.05% and 0.10%. Five nanometers carbon coating were used to ensure sample conductivity.

## 3. Results and Discussions

### 3.1. Dispersion Process

**Figure 3:**
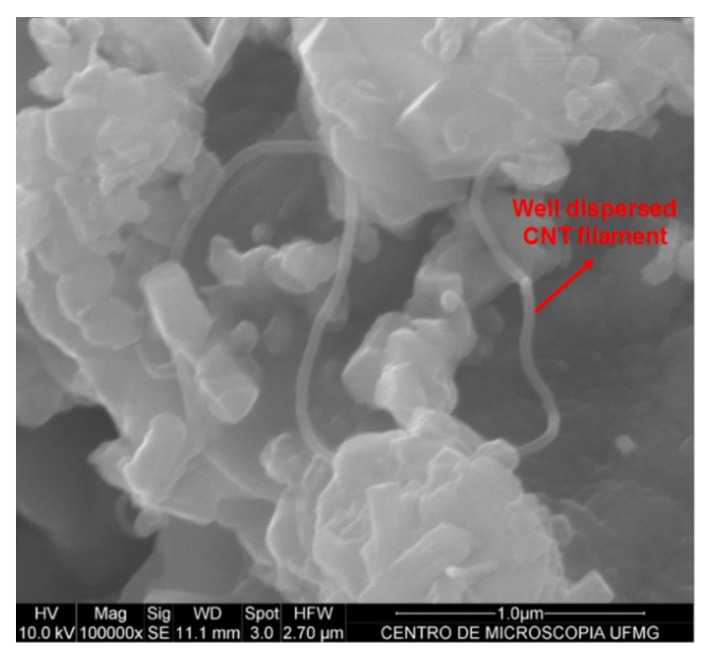
Scanning electron microscopy images of the anhydrous cement with 0.05% of CNTs.

**Figure 4:**
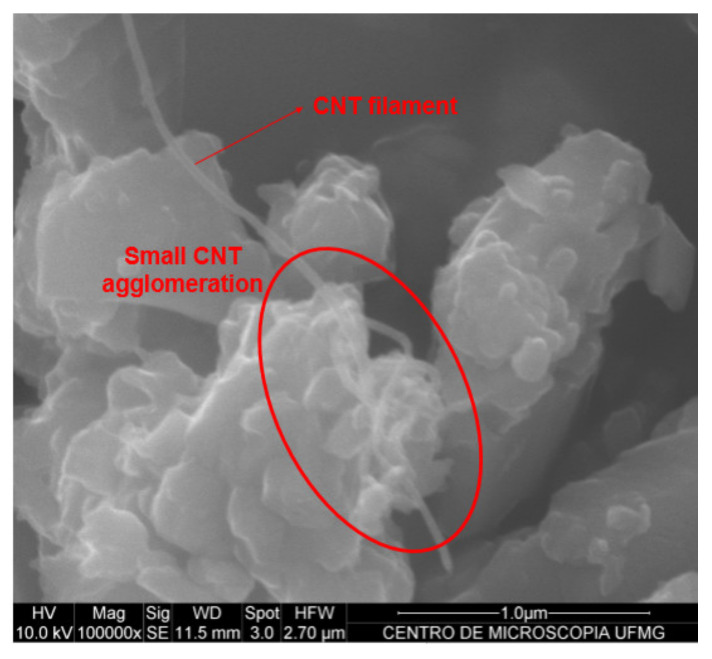
Scanning electron microscopy images of the anhydrous cement with 0.10% of CNTs with small agglomeration.

Figure 3 and Figure 4 show the efficiency of CNT dispersion on cement particles, evaluated by scanning electron microscopy (using a FEG Quanta 200 made by FEI Company equipment located at the Center of Microscopy, UFMG, Belo Horizonte, Minas Gerais, Brazil). The first Figure displays the cement with 0.05% of dispersed CNTs by weight of cement (bwoc), while the second Figure displays the cement with 0.10% CNTs bwoc.

The CNT filaments are well dispersed in the anhydrous cement with 0.05% concentration of CNTs (see Figure 3). The cement particles with 0.10% of CNTs (Figure 4) also show well-dispersed CNTs on the surface of the cement particles, but small agglomerations are present. Figure 3 and Figure 4 show that the effective CNT dispersion limit for this specific methodology is close to 0.05%, since adding 0.10% of CNTs, small agglomerations are already observed.

The results of the surface area by multipoint nitrogen adsorption technique and the ratio of CNTs dispersed on the anhydrous cement surface area are presented in Table 2, and as expected, the presence of CNTs increased the surface area due to their high specific surface area. Incorporating 0.05% of CNTs, the surface area increased 4.4%, whereas the incorporation of 0.10% recorded an increase of 17.7% as compared with the reference (REF-ISO). The CNT/cement surface area rate was recorded to identify the ideal proportion for CNT filaments to disperse between anhydrous cement particles. According to Figure 3 and Figure 4, CNTs in both proportions of 0.89 and 1.78, respectively, seem to be well dispersed, however, 0.10% of CNTs bwoc (1.78 g/m^2^/g) caused some agglomeration.

According to the results of Rocha and Ludvig (2018) [9], considering the compressive and tensile strengths, the best result achieved by the dispersion of CNTs in isopropanol, was close to 0.05% bwoc. This implied that the optimal CNT ratio dispersible by that methodology was close to 0.89 g of CNTs per m^2^·g^−1^ of anhydrous cement surface area and a superior mechanical performance in cement pastes prepared with anhydrous cement with 0.05% of CNTs was expected.

### 3.2. Evaluation of the Mechanical Behavior

**Figure 5:**
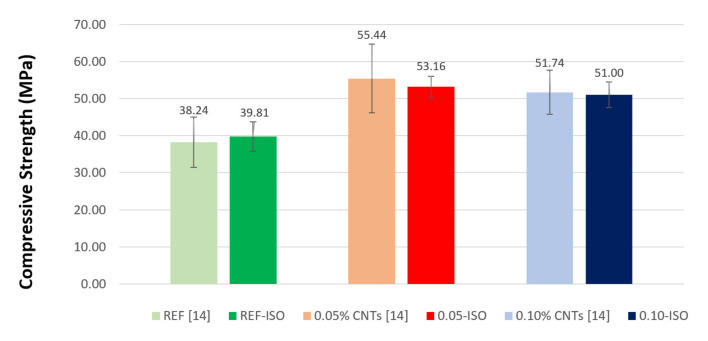
Compressive strength test results as compared with previous results.

The results of compressive strength are presented in Figure 5; it is compared with the results achieved by Rocha and Ludvig (2018) [9]. These tests were performed on 5 cm diameter and 10 cm height cylindrical samples (columns on the left side) to determine compressive and splitting tensile strengths, meanwhile, the columns on the right side demonstrate the results of compressive strength determined by the half-prism and flexural tensile strength tests (as described in Section 2.3).

The cement paste in the presence of 0.05% and 0.10% of CNTs recorded strength gains of 34% and 28%, respectively, values close to those obtained by Rocha and Ludvig (2018) [9] who recorded 45% and 35%. On the one hand, according to the statistical analysis of variance (ANOVA), the difference between the compressive strength averages of REF, 0.05-ISO and 0.10-ISO pastes can be considered as significant. These gains suggest a reinforcement effect of cement pastes in these CNT proportions. On the other hand, also by ANOVA, the difference between the results for the compressive strength averages in the present work and the results presented by Rocha and Ludvig (2018) [9] are not identified as significant.

**Figure 6:**
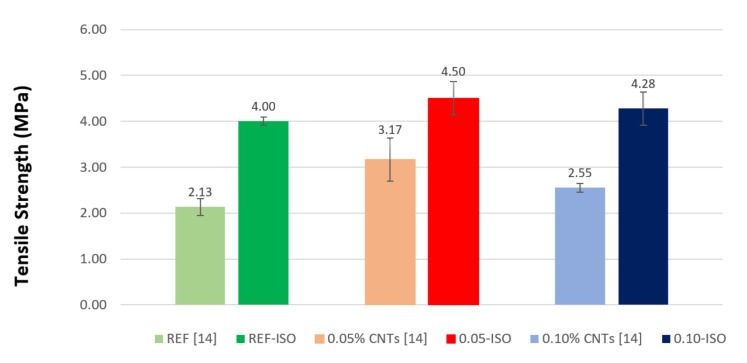
Tensile strength results as compared with previous results.

The flexural tensile strength results are presented in Figure 6. The compressive strength results are similar to those obtained by Rocha and Ludvig (2018) [9]. In addition to the similar tendencies observed earlier, the flexural tensile strength results are almost two times higher than the splitting tensile strength recorded by the cited authors [9]. The difference can be explained by the fact that flexural tests are more conservative because of the two-dimensional state of stress and the size effect [29,30,31].

Cement pastes incorporating 0.05% and 0.10% of CNTs recorded gains of 12% and 7%, respectively, whereas Rocha and Ludvig (2018) [9] recorded 49% and 20%. According to ANOVA, the difference between the averages of tensile by flexural strength is not significant. However, the gains imply a reinforcement of cement pastes as an effect of the addition of predispersed CNTs.

The mechanical characterization by compressive and tensile tests recorded a more expressive increase in cement pastes adding 0.05% of CNTs, which suggests efficient dispersion of the nanomaterial in cement particles by the adopted methodology under this concentration and a possible negative effect at 0.10% addition due to agglomeration.

**Figure 7:**
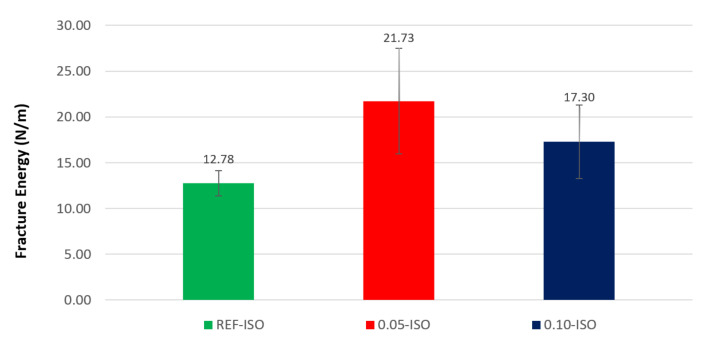
Fracture energy results.

The results of the fracture energy tests are presented in Figure 7. Cement pastes incorporating 0.05% and 0.10% of CNTs recorded gains of 70% and 35%, respectively. Although the cement paste with 0.05% of CNTs recorded a relatively high standard deviation, the lowest result of fracture energy in the cement paste with carbon nanotubes is still higher than the highest result of the reference paste. According to the ANOVA test, the difference between the fracture energy averages is not significant.

**Figure 8:**
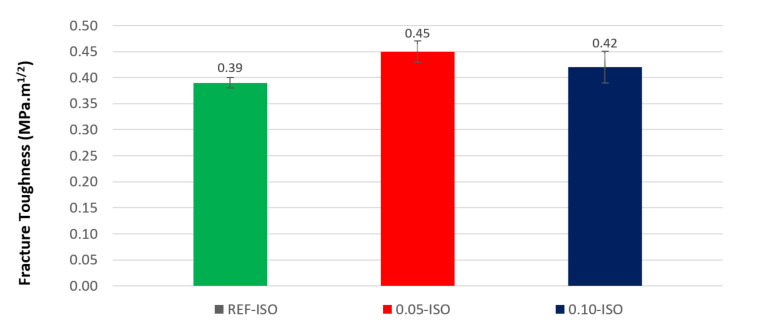
Fracture toughness results.

**Figure 9:**
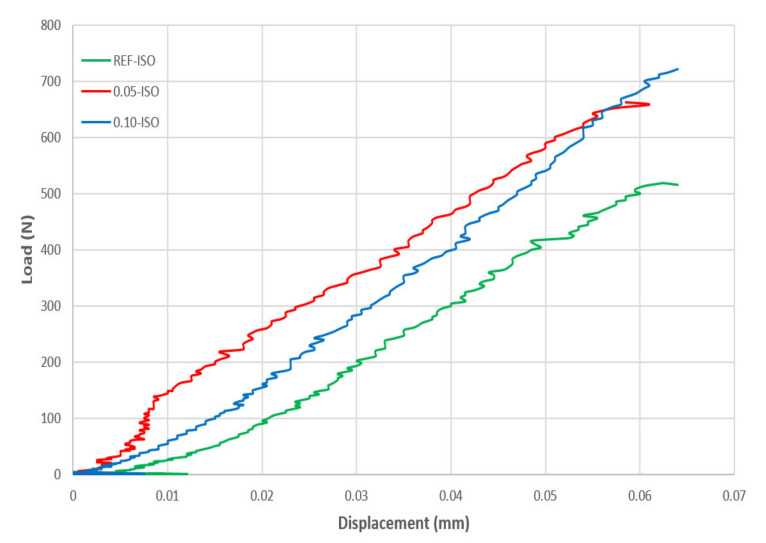
Three-point flexural test load-displacement diagrams.

The results of the fracture toughness test are presented in Figure 8. Cement pastes incorporating 0.05% and 0.10% of CNTs recorded gains of 14% and 7%, respectively. According to ANOVA, likewise it is not possible to state that the difference between the averages of fracture toughness is significant, as pointed out in the case of flexural strength and fractural energy. The results of the load-displacement curve integration are presented in Table 3 and the load-displacement diagram of the most representative curve of each cement paste formulation is presented in Figure 9. The results, as presented in the diagram, were extracted from the three-point flexural tests, which were conducted until failure according to the adopted methodology (actuator controlled by vertical displacement and deformations recorded by DIC).

The fracture energy considering the curve integration area, according to the results obtained by Equation (1), in which a better performance of 0.05-ISO could be observed. This result is expected once the formulation in Equation (1), considering the curve integration area of the load-displacement diagram. The standard deviations of the curve integration areas were 0.0011, 0.0022, and 0.0023 N·m for REF-ISO, 0.05-ISO, and 0.10-ISO, respectively.

The maximum gains in fracture energy and fracture toughness were obtained in the presence of 0.05% of CNTs, which were in accordance with the results of the compression and tensile tests, and suggested that, in such proportions, the hydration products adhered well to the nanomaterial and caused a refinement of the capillary pores [8] and crack control at the submicron level [23]. These results could be related to the effective dispersion of carbon nanotubes that permitted it to act as a fibrous reinforcement and allow greater load application and deformations of the samples before the rupture.

### 3.3. Evaluation of the Physical Parameters

**Figure 10:**
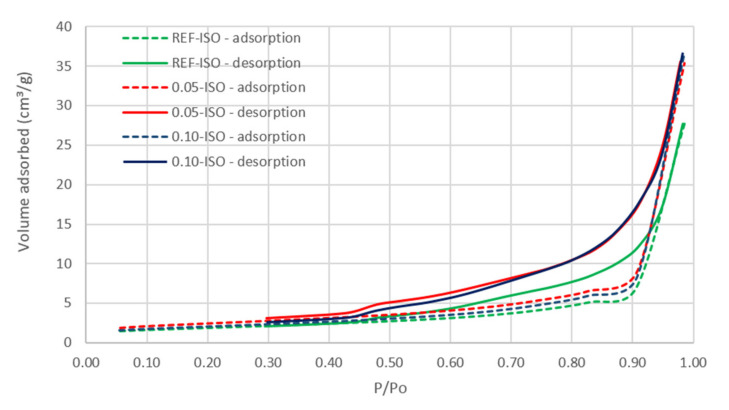
Isotherm curves of cement pastes.

**Figure 11:**
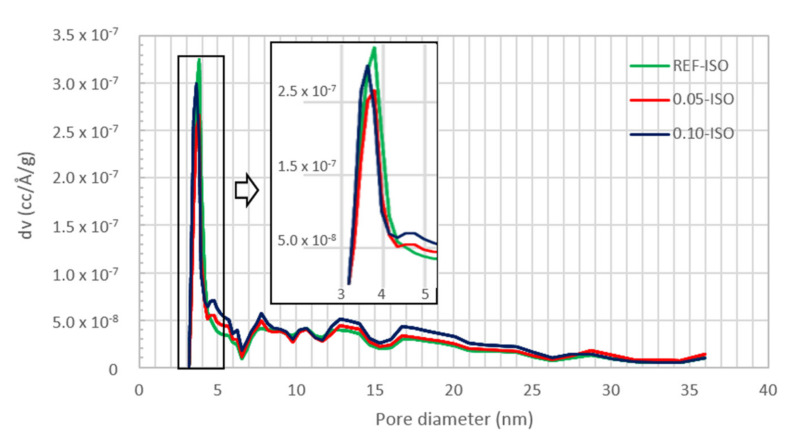
Pore size distribution of cement paste obtained by the density functional theory (DFT) method.

**Figure 12:**
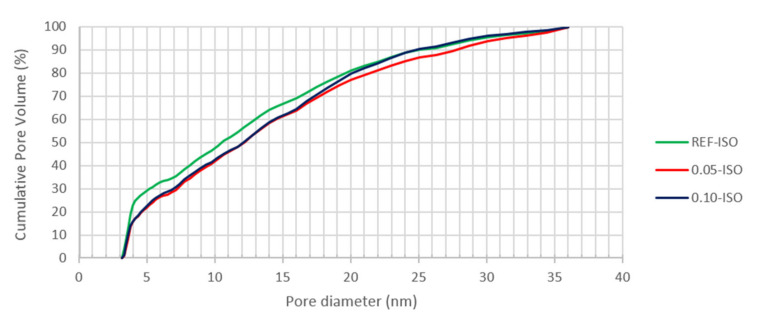
Cumulative pore volume of cement pastes obtained by the DFT method.

The nitrogen adsorption and desorption isotherms and the pore distribution results by DFT analysis are displayed in Figure 10, Figure 11 and Figure 12, i.e., isotherm, pores diameter results, and pore volume results, respectively.

The isotherm curves of these three cement pastes are similar. According to the International Union of Pure and Applied Chemistry (IUPAC), the classification of the three isotherms presented in Figure 10 is isotherm type II and hysteresis type H3, which indicate slit shape pores, characteristics of C–S–H [32].

According to Figure 10, a higher hysteresis in the presence of CNTs is observed; the desorption curve is more distant from the adsorption for 0.05-ISO and 0.10-ISO, than for the REF-ISO paste. This behavior is related to the difficulty of desorbing the gas molecules that are condensed in the smaller pores identified in cement pastes prepared with CNTs, suggesting pore refinement.

The observed type II isotherm is related to the low degree of pore curvature and the structure [33] corresponding to the characteristics of cement composites.

The pore size distribution graphs obtained by DFT technique were used for the analysis of the pores in the mesoporous region, with diameters between 2 and 50 nm. The results presented in Figure 10 and Figure 11 indicate a higher volume of fine pores in the REF-ISO as compared with cement pastes with CNTs.

According to the results presented in Figure 12, 0.05-ISO and 0.10-ISO have a smaller quantity of pores, up to 20 nm in diameter as compared with REF-ISO. However, the results presented in Figure 10 suggest pore size reduction of 0.05-ISO and 0.10-ISO, which presented difficulty in desorbing the gas. A justification for the desorption difficulty, despite the pore reduction, is the reduction of the specific pores larger than 20 nm in cement pastes with CNTs. These results are in agreement with Xu, Liu and Li (2015) [15], who argued that the presence of CNTs reduced the quantity of pores with diameters larger than 50 nm which were considered to be harmful to the mechanical properties of the cement composites, and increased the quantity of pores smaller than 50 nm. Thereby, the BET results suggest that the presence of CNTs leads to a reduction in pores larger than 20 nm in diameter and an increase in the quantity of pores larger than 20 nm. The reduction of pores that are considered to be harmful contributes to the improvement of the mechanical properties recorded in this work.

The results shown in Figure 12 indicate that 20% of the total pore volume, up to 40 nm (mesoporous range), is higher than the following: (i) 19.2 nm in REF-ISO, (ii) 20.1 nm in 0.05-ISO, and (iii) 21.2 nm in 0.10-ISO. Considering 30% of the total pore volume diameter, the REF-ISO pores are smaller than 5 nm and the 0.05-ISO and 0.10-ISO pores are smaller than approximately 7 nm. These data indicate similarity in the pore distribution of 0.05-ISO and 0.10-ISO pastes and a difference between REF-ISO and pastes prepared with CNTs. The presence of CNTs affected the cement pastes nanostructure resulting in a lower percentage of finer pores, according to Nochaiya and Chaipanich (2010) [14]. As a consequence, cement pastes reinforced with CNTs apparently have a denser matrix, resulting in superior mechanical properties. An explanation for this result could be the CNTs’ ability to cause nucleation of the cement hydration products, forming denser structure of the cement paste at the nanoscopic level.

It is noteworthy that the DFT analysis covered pores up to 40 nm and complementary to the nanoscale pore distribution, water absorption, void index, and density of the saturated and dry samples obtained by water absorption technique are presented in Table 4.

Table 4 demonstrates that the reference presented elevated pore volume obtained by water absorption. Water absorption and void index of the 0.05-ISO and 0.10-ISO pastes exhibit a certain decrease, meanwhile, the density (both saturated and dry) of these samples increase as compared with the reference. These results indicate a lower level of porosity of cement pastes with CNT addition. Furthermore, the water absorption, void index, and density data are consistent with mechanical behavior, as porosity is inversely related to compressive strength. Complementing these results, the pore distribution graphs obtained by the DFT technique analyzed the mesopores and recorded that the REF-ISO presented a higher percentage of finer pores. It can be concluded that CNTs affect the pore structure of the cement paste at both scales, reducing the porosity of the C-S-H and the paste. These findings reinforce the hypothesis that CNTs have a nucleating effect on cement hydration products, contributing to the reduction of small pores in this diameter pores range.

**Figure 13:**
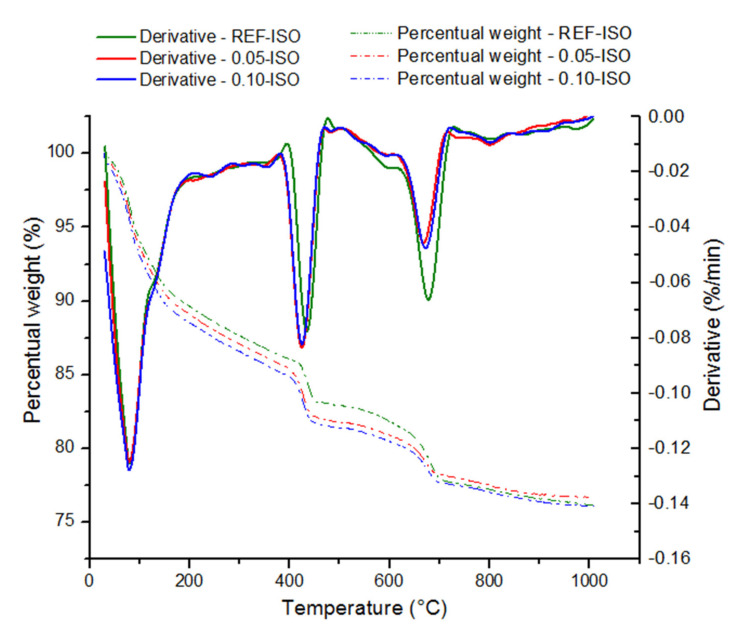
Results of the thermogravimetric analysis.

The results of thermogravimetric analysis are exhibited in Table 5 and Figure 13.

The ranges indicated in Table 5 are based on the mass loss peaks observed in the derivative thermogravimetric curves. The weight losses recorded are due to the following: (i) up to 150 °C, dehydration of water pore; (ii) from 150 °C to 400 °C, dehydration of different stages of C–S–H; (iii) from 400 °C to 600 °C, dehydroxylation of CH; and (iv) between 600 °C and 1000 °C, decarbonation of CaCO_3_ [34,35,36]. There are different concepts on the temperature range of cement paste hydration product decomposition and it is especially challenging to establish a boundary between the water pore and C–S–H decomposition because of the hydrophilic behavior of C-S-H surface [37], therefore, the separation was based on the onset and offset of the mass loss peaks recorded.

The thermogravimetric analysis resulted in similar curve shapes, indicating the decomposition of the same hydration products in the three analyzed samples. The total weight loss was 20.3% for REF-ISO, 19.9% for 0.05-ISO, and 20.4% for 0.10-ISO.

The above results indicate a higher percentage of C−S−H and CH for 0.05-ISO and 0.10-ISO compositions, respectively, which could be explained by the densification of hydration products due to the nucleating effect of CNTs. The third temperature range (600 °C to 1000 °C) corresponds to the calcium carbonate (CaCO_3_) decomposition and, in this range, the REF-ISO shows a higher amount of CaCO_3_ as compared with 0.05-ISO and 0.10-ISO. This compound is the result of the carbonic acid (H_2_CO_3_) reaction, which is formed in the presence of carbon dioxide (H_2_CO_3_) and humidity (H_2_O) in the cement matrix. This result is in accordance with the water absorption test results. The higher pore volume in the REF-ISO would permit higher permeability of CO_2_, contributing to a more pronounced formation of CaCO_3_. The porosity reduction with the addition of CNTs could have occurred because it acts as nucleation sites for hydration products, resulting in C–S–H densification, as suggested by Makar and Chan (2009) [22] and displayed in Table 5. The thermogravimetric analysis evidenced that CNTs in the proportion of 0.05% and 0.10% affect the microstructure of cement pastes, once 0.05-ISO and 0.10-ISO presented higher relative quantity of hydration products (CH and C–S–H) and smaller quantity of CaCO_3_.

**Figure 14:**
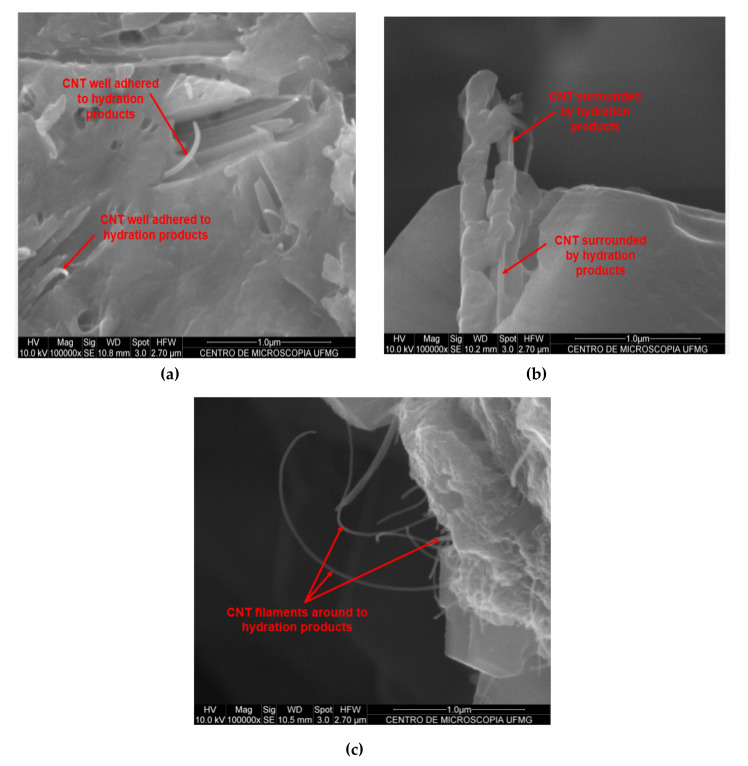
(**a**,**b**) Scanning electron microscopy images of hydrated cement pastes with 0.05% of CNTs (0.05-ISO); (**c**) Scanning electron microscopy images of hydrated cement pastes with 0.10% of CNTs (0.10-ISO).

The scanning electron microscopy images of cement pastes with CNTs are shown in Figure 14. Figure 14a,b shows hydrated cement paste with 0.05% of CNTs bwoc and Figure 14c shows hydrated cement paste with 0.10% of CNTs bwoc.

In the images of cement paste fragments with 0.05% of CNTs (Figure 14a,b), it is difficult to identify CNTs. This fact could indicate that the CNTs were well dispersed and well incorporated in the cement hydration products. The hydration products could have hidden well-dispersed CNTs. The small filaments highlighted in Figure 14a can be identified as fragmented CNTs, evidencing good interaction with cement hydration products. Figure 14b displays a well-adhered CNT filament surrounded by hydration products, confirming that this nanomaterial acts as a nucleation site for cement hydration. In the 0.10-ISO paste, Figure 14c, CNTs are easier to visualize, which could indicate lower interaction with hydration products and worse dispersion as compared with 0.05-ISO.

In the proportion of 0.05% of CNTs, the maximum strength gain was recorded and the scanning electron microscopy images indicated better CNT dispersion. Otherwise, in the proportion of 0.10% of CNTs, although the strength gains recorded were compared to REF-ISO, there were indications that the effective dispersion limit was exceeded, resulting in agglomerations that possibly acted as strength concentration and contributed to lower strength as compared with 0.05-ISO.

## 4. Conclusions

The use of MWCNTs dispersed in non-aqueous isopropanol media on the surface of anhydrous cement particles was discovered to be an effective way for cement paste nanocomposite preparation. Nanostructured cement pastes in the proportions of 0.05% and 0.10% of CNTs in relation to the cement weight revealed higher compressive strength, flexural tensile strength, fracture energy, and fracture toughness. The gains obtained could evidence that CNTs act as nucleation of cement hydration products and fibrous reinforcement in cement-based material by this dispersion process.

The cement pastes with CNTs presented denser structure according to the water absorption test results, suggesting general pore refinement. The porosimetry analysis of nitrogen condensation in the mesoporous range indicated a lower quantity of finer pores (below 10 nm) in the presence of CNTs. These results are related to the CNTs’ behavior as nucleation sites, supported by scanning electron microscopy images, where it is observed that the CNTs’ filaments are surrounded and adhered to hydration products. Moreover, the thermogravimetric analysis also indicated that, in the presence of CNTs, the quantity of C-S-H and CH in the hydrated cement pastes was higher, corroborating with the statement that CNTs can additionally perform as nucleation sites of cement hydration products, resulting in a denser matrix with pore refinement. Additionally, the higher amount of carbonated material of REF paste indicates a reduction of permeability as an effect of CNT addition.

The comparison of the different concentrations demonstrated that the best result was achieved in the presence of 0.05% of CNTs. By the described methodology, the effective dispersion limit was reached in this proportion and in the ratio close to 0.89 g of CNTs per m^2^ g^−1^ of anhydrous cement surface. The scanning electron microscopy images suggested that the amount of 0.10% of CNTs permitted the formation of small agglomerations that possibly contributed to lower strength regarding the paste with 0.05% CNT content.

## Figures and Tables

**Table 1:** Cement paste formulations.

Identification	Formulation
REF-ISO	Cement paste prepared without CNTs
0.05-ISO	Cement paste prepared with addition of 0.05% of CNTs
0.10-ISO	Cement paste prepared with addition of 0.10% of CNTs

**Table 2:** Surface area obtained by nitrogen adsorption.

Identification	Surface Area (m^2^ g^−1^)	CNTs / Cement Surface Area Rate (g m^−^^2^/g)
Anhydrous Cement	1.687	-
Cement + 0.05% CNT	1.761	0.89
Cement + 0.10% CNT	1.986	1.78

**Table 3:** Results of the load-displacement curve integration.

Sample	REF-ISO		0.05-ISO		0.10-ISO	
	Area [N.m]	Average (N·m)	Area (N·m)	Average (N.m)	Area (N·m)	Average (N·m)
Specimen 1	0.0174745	0.015821167	0.02981125	0.026502003	0.01956575	0.021834167
Specimen 2	0.01526125		0.02421901		0.0252675	
Specimen 3	0.01472775		0.02547575		0.02066925	

**Table 4:** Water absorption results.

Identification	Water Absorption (%)	Void Index (%)	Density of Saturated Samples (g cm^−3^)	Density of Dried Samples (g cm^−3^)
REF-ISO	20.90%	34.03%	1.97	2.47
0.05-ISO	18.48%	31.96%	2.05	2.54
0.10-ISO	19.23%	32.76%	2.03	2.53

**Table 5:** Mass loss recorded by thermogravimetric analysis.

Mass Loss of Each Cement Paste by Temperature Range							
Temperature Range	Decomposition	REF -ISO		0.05-ISO		0.10-ISO	
		Mass (mg)	Mass Loss (%)	Mass (mg)	Mass Loss (%)	Mass (mg)	Mass Loss (%)
30 °C to 150 °C	Water pore	0.341	33.83	0.380	37.34	0.400	39.17
150 °C to 400 °C	Water pore and C–S–H	0.232	22.95	0.242	23,81	0.234	22.92
400 °C to 600 °C	CH	0.192	19.01	0.220	21.60	0.207	20.24
600 °C to 1000 °C	CaCO_3_	0.244	24.22	0.175	17.25	0.180	17.67

## References

[1] Makar J.M., Beaudoin J.J. (2004). Carbon nanotubes and their application in the construction industry. Spec. Publ.-R. Soc. Chem..

[2] Collins F., Lambert J., Duan W.H. (2012). The influences of admixtures on the dispersion, workability, and strength of carbon nanotube–OPC paste mixtures. Cem. Concr. Compos..

[3] Hawreen A., Bogas J., Guedes M., Pereira M.F.C. (2018). Dispersion and reinforcement efficiency of carbon nanotubes in cementitious composites. Mag. Concr. Res..

[4] Rashad A.M. (2017). Effect of carbon nanotubes (CNTs) on the properties of traditional cementitious Materials. Constr. Build. Mater..

[5] Al-Rub R.K.A., Ashour A.I., Tyson B.M. (2012). On the aspect ratio effect of multi-walled carbon nanotube reinforcements on the mechanical properties of cementitious nanocomposites. Constr. Build. Mater..

[6] Hu Y., Luo D., Li P., Li Q., Sun G. (2014). Fracture toughness enhancement of cement paste with multi-walled carbon nanotubes. Constr. Build. Mater..

[7] Wang B., Han Y., Liu S. (2013). Effect of highly dispersed carbon nanotubes on the flexural toughness of cement-based composites. Constr. Build. Mater..

[8] Zou B., Chen S.J., Korayem A.H., Collins F., Wang C.M., Duan W.H. (2015). Effect of ultrasonication energy on engineering properties of carbon nanotube reinforced cement pastes. Carbon.

[9] Rocha V.V., Ludvig P. (2018). Nanocomposites prepared by a dispersion of CNTs on cement particles. Archit. Civ. Eng. Environ..

[10] Li G.Y., Wang P.M., Zhao X. (2005). Mechanical behavior and microstructure of cement composites incorporating surface-treated multi-walled carbon nanotubes. Carbon.

[11] Hawreen A., Bogas J., Dias A. (2018). On the mechanical and shrinkage behavior of cement mortars reinforced with carbon nanotubes. Constr. Build. Mater..

[12] Rocha V.V., Ludvig P., Trindade A.C.C., de Andrade Silva F. (2019). The influence of carbon nanotubes on the fracture energy, flexural and tensile behavior of cement based composites. Constr. Build. Mater..

[13] Carriço A., Bogas J.A., Hawreen A., Guedes M. (2018). Durability of multi-walled carbon nanotube reinforced concrete. Constr. Build. Mater..

[14] Nochaiya T., Chaipanich A. (2011). Behavior of multi-walled carbon nanotubes on the porosity and microstructure of cement-based materials. Appl. Surf. Sci..

[15] Xu S., Liu J., Li Q. (2015). Mechanical properties and microstructure of multi-walled carbon nanotube-reinforced cement paste. Constr. Build. Mater..

[16] Isfahani F.T., Li W., Redaelli E. (2016). Dispersion of multi-walled carbon nanotubes and its effects on the properties of cement composites. Cem. Concr. Compos..

[17] Souza Filho A.G., Fagan S.B. (2007). Funcionalização de nanotubos de carbono. Quím. Nova.

[18] Liu Y., Gao L., Sun J. (2007). Noncovalent functionalization of carbon nanotubes with sodium lignosulfonate and subsequent quantum dot decoration. J. Phys. Chem. C.

[19] Batiston E.R., Hampinelli D., Oliveira R.C., Gleize P.J.P. (2010). Funcionalização e efeito da incorporação de nano tubos de carbono na cinética de hidratação em matrizes cimentícias. Congr. Bras. Concr..

[20] Ludvig P., Calixto J.M., Ladeira L.O., Gaspar I.C. (2011). Using converter dust to produce low cost cementitious composites by in situ carbon nanotube and nanofiber synthesis. Materials.

[21] Alsharefa J.M., Tahaa M.R., Khan T.A. (2017). Physical dispersion of nanocarbons in composites—A review. Technol. J..

[22] Makar J.M., Chan G.W. (2009). Growth of cement hydration products on single-walled carbon nanotubes. J. Am. Ceram. Soc..

[23] Makar J., Margeson J., Luh J. Carbon nanotube/cement composites-early results and potential applications. Proceedings of the 3rd International Conference on Construction Materials: Performance, Innovation and Structural Implications.

[24] Silva R.A., Guetti P., Da Luz M.S., Rouxinol F., Gelamo R.V. (2017). Enhanced properties of cement mortars with multilayer graphene nanoparticles. Constr. Build. Mater..

[25] Dally J.W., Riley W.F. (1991). Experimental Stress Analysis.

[26] American Society for Testing Materials (2002). ASTM 349-02. Standard Test Methods for Compressive Strength of Hydraulic-Cement Mortars (Using Portions of Prisms Broken in Flexure).

[27] ASTM International (2013). ASTM C642-13. Standard Test Method for Density, Absorption, and Voids in Hardened Concrete.

[28] Korpa A., Trettin R. (2006). The influence of different drying methods on cement paste microstructures as reflected by gas adsorption: Comparison between freeze-drying (F-drying), D-drying, P-drying and oven-drying methods. Cem. Concr. Res..

[29] Balbo J.T. (2013). Relações entre resistências à tração indireta e à tração na flexão em concretos secos e plásticos. Rev. IBRACON Estrut. Mater..

[30] Bažant Z.P. (2000). Size effect. Int. J. Solids Struct..

[31] Hu X., Duan K. (2008). Size effect and quasi-brittle fracture: The role of FPZ. Int. J. Fract..

[32] Ludvig P., Calixto J.M.F., Ladeira L.O., Souza T.C., Paula J.N. (2017). Analysis of Cementitious Composites Prepared with Carbon Nanotubes and Nanofibers Synthesized Directly on Clinker and Silica Fume. J. Mater. Civ. Eng..

[33] Sing K.S., Williams R.T. (2004). Physisorption hysteresis loops and the characterization of nanoporous materials. Adsorpt. Sci. Technol..

[34] Fordham C.J., Smalley I.J. (1985). A simple thermogravimetric study of hydrated cement. Cem. Concr. Res..

[35] Almeida A.E.F.D.S., Tonoli G.H.D., Santos S.F.D., Savastano H. (2013). Improved durability of vegetable fiber reinforced cement composite subject to accelerated carbonation at early age. Cem. Concr. Compos..

[36] Ma Q., Guo R., Zhao Z., Lin Z., He K. (2015). Mechanical properties of concrete at high temperature—A review. Constr. Build. Mater..

[37] Bonnaud P.A., Ji Q., Coasne B., Pellenq R.M., Van Vilet K.J. (2012). Thermodynamics of water confined in porous calcium-silicate-hydrates. Langmuir.

